# Temperature-regulated expression of outer membrane proteins in Shigella flexneri

**DOI:** 10.1186/1757-4749-5-38

**Published:** 2013-12-11

**Authors:** Hemavathy Harikrishnan, Asma Ismail, Kirnpal-Kaur Banga Singh

**Affiliations:** 1 Department of Medical Microbiology & Parasitology, School of Medical Sciences, Health Campus, Universiti Sains Malaysia, 16150 Kubang Kerian, Kelantan, Malaysia; 2 Institute for Research in Molecular Medicine (INFORMM), Universiti Sains Malaysia, 16150 Kubang Kerian, Kelantan, Malaysia; 3 Islamic Science Institute, Islamic Science University of Malaysia, 71800 Nilai, Negeri Sembilan, Malaysia

## Abstract

**Background:**

Bacteria exist widely in a diversity of natural environments. In order to survive adverse conditions such as nutrient depletion, biochemical and biological disturbances, and high temperature, bacteria have developed a wide variety of coping mechanisms. Temperature is one of the most important factors that can enhance the expression of microbial proteins. This study was conducted to investigate how outer membrane proteins (OMPs) of the bacterium *Shigella flexneri* respond to stress, especially during fever when the host’s body temperature is elevated.

**Methods:**

OMPs of *S. flexneri* ATCC 12022 and clinical isolate SH057 were extracted from an overnight culture grown at 37, 38.5, and 40°C. Comparisons of the expressed proteins under the different growth conditions were based on equal numbers of bacterial cells loaded in the SDS-PAGE gels. Separated proteins were stained with Coomassie brilliant blue. Selected proteins showing increased expression at 38.5 and 40°C were characterized by performing MALDI-ToF-ToF.

**Results:**

Different degrees of expression were demonstrated for different proteins expressed at 37°C compared to 38.5 and 40°C*.* The proteins with molecular sizes of 18.4, 25.6, and 57.0 kDa showed increased expression level at increasing temperature and were identified as Dps, WrbA, and PepA, respectively.

**Conclusion:**

This study revealed that strains of *S. flexneri* respond at the proteomic level during stress caused by elevated temperature by decreasing the expression of proteins, maintaining the level of important proteins, or enhancing the levels of proteins presumably involved in survival and virulence.

## Introduction

Bacillary dysentery (also known as shigellosis) caused by *Shigella* spp. is a public health concern in developing countries and for travelers from industrialized nations [1]. The annual numbers of shigellosis episodes and deaths in Asia were estimated to be 91 million and 414,000, respectively, with *S. flexneri* and *S. sonnei* being the first and second most common serotypes [2]. A 9-year retrospective study conducted in Northeast Malaysia [3] showed that the isolation rate is 9.99% of the total bacterial pathogens isolated from stool specimens, with *S. sonnei* and *S. flexneri* being the most common species isolated Shigellosis causes diarrhea or dysentery with frequent mucoid bloody stools, abdominal cramps, and fever [4]. The presentation of these clinical symptoms is due to the invasion and growth of the bacterium in the intestinal mucosal cells. The bacterium subsequently causes cell death and spreads laterally to infect and kill the adjacent epithelial cells. This mechanism can lead to mucosal ulceration, inflammation, and bleeding [5]. The life-threatening complications caused by shigellosis include metabolic derangements, such as dehydration, hyponatraemia, hypoglycaemia, hypoproteinaemia, and severe anorexia, as well as intestinal complications such as toxic megacolon, rectal prolapse, and intestinal perforation [6,7]. Bacteraemia due to *Shigella* is relatively rare but does occur [8,9].

The outer membrane of Gram negative bacteria plays many roles in cellular function in addition to the classical role of transporting ions through the porin proteins [10]. Protective immunity elicited by outer membrane proteins (OMPs) has been documented for several Gram negative organisms [11]. During the course of infection, growth of bacteria is influenced by various environmental factors, including pH, osmotic strength, oxygen, iron availability, and temperature, which may change dramatically. Thus, the bacteria must adapt to and survive in the new environment [12]. Hale (1991) found that some temperature-sensitive *Shigella* spp. are capable of invading the intestinal epithelium only at elevated temperature and that invasion does not take place at 37°C [13]. Ellis (1996) reported that certain proteins are overexpressed in response to high temperature (e.g., heat shock proteins (HSPs) and serve as defense mechanisms against various environmental stresses [14]. Research has also shown that the HSPs are usually related to the virulence of the pathogens [15].

In this study, we compared the OMP expression profiles of *S. flexneri* cells grown at 37, 38.5, and 40°C to evaluate expression level changes in cells that are induced by temperature increase. Those proteins that showed a prominent increase in expression at 38.5 and 40°C were subsequently identified using MALDI-ToF-ToF analysis in order to demonstrate the presence of HSPs.

## Materials and methods

### Shigella strains and the sereny test

The four clinical strains (SH052, SH057, SH060, and SH062) of *Shigella flexneri* 2a used in this study were obtained from the Department of Medical Microbiology and Parasitology, School of Medical Sciences, Universiti Sains Malaysia. Virulence of these isolates was checked by performing Sereny test in order to determine their ability to cause keratoconjunctivitis in guinea pigs. This animal study was conducted in accordance with the requirements of the Animal Ethics Committee, Universiti Sains Malaysia (AECUSM) approval protocol PPSG/07(A)/044.

The four *S. flexneri* strains were tested via inoculation into guinea pig eyes as follows. The bacterial strains were grown overnight in LB broth. They were used for the test when they reached 10^8^ CFU in normal saline (0.9%), as determined by measuring optical density (OD) at 600 nm. Each eye of a Hartley guinea pig (n = 6) was inoculated in the conjunctival sac with 10^8^ CFU of one of the wild strains. Guinea pigs were examined daily for 5 days, and their inflammatory responses were graded according to Hartman *et al.*[16]. Development of the disease was rated as follows: 0: no disease or mild; 1: mild conjunctivitis or late development and/or rapid clearing of symptoms; 2: keratoconjunctivitis without purulence; and 3: fully developed keratoconjunctivitis with purulence. Among the four strains, SH057 caused fully developed keratoconjunctivitis with purulence in guinea pigs and thus was used in this study. A reference strain, *S. flexneri* ATCC 12022, was the standard organism used when performing protein profiling in this study.

*S. flexneri* ATCC 12022 and the clinical isolate were maintained in nutrient slants agar and Tryptic soy broth containing 20% glycerol. Working cultures were prepared by inoculating one single colony in 10 ml of nutrient broth, which was then incubated overnight at 37°C with shaking at 200 rpm in an orbital shaker (Forma Orbital Shaker, Model-420, USA). The purity of the culture was determined by inoculating it on blood agar. The identities of both *S. flexneri* ATCC 12022 and the clinical isolate were confirmed by performing standard biochemical identification using triple sugar iron agar, sulfide-indole motility medium, urease, methylene red, and citrate.

### Outer membrane protein (OMP) preparation

*S. flexneri* ATCC 12022 and clinical isolate SH057 were grown overnight in nutrient broth at 37, 38.5, and 40°C under shaking at 200 rpm in separate orbital shakers (Forma Orbital Shaker). Preparation of the OMPs expressed at the three temperatures was performed following the published procedure for extracting OMPs of *Salmonella typhi*[17]. Cells were harvested by centrifugation at 15,900 x g for 18 minutes and resuspended in 8 ml of 0.01 M HEPES (N-2 hydroxy ethyl piperazine-N’-2ethane sulfonicacid) buffer (pH 7.4). This suspension was then mixed with 8 μl of 10 mM DNAse (Sigma, USA), 8 μl of 10 mM RNAse (Sigma, USA), and 800 μl of 100 mM phenylmethylsulfonyl fluoride (Calbiochem, USA). Bacterial cell in the suspensions were disrupted by vortexing (HeidolphReax Top, Germany) with glass beads (~0.2 mm in diameter, BDH Chemical Ltd., UK) for 1.5 hours, with 1 minute alternate on ice until 95% lysis was achieved. Cell disruption was confirmed using the Gram staining method. The cell lysate obtained was aspirated and the glass beads were washed with 0.01 M HEPES buffer until the cell turbidity was clear. The unlysed cells were removed by centrifugation using a high-speed refrigerated centrifuge (Kubota, Model 6930, Japan) at 7,800 × g at 4°C for 15 minutes. The supernatant was then centrifuged with an ultracentrifuge (Hitachi, Model CP 80MX, Japan) at 145,100 × g at 4°C for 1 hour (using rotor type P40 ST) to obtain crude cell envelopes. The Triton X-100 extraction method was used to separate the inner and outer membranes. The pellet containing the crude envelopes was treated with 0.01 M HEPES containing 4% Triton X-100 (Bio-Rad, USA) to solubilize the inner membrane. The mixture was incubated at room temperature for 10 minutes. The insoluble OMPs were pelleted using the ultracentrifuge at 181,800 × g at 4°C for 1 hour (using rotor type P55 ST2). The pellet was resuspended with 4 ml of 30 mM Tris–HCl, pH 8.0 and stored at −20°C until use.

### Profiling of the OMP(s) of S. flexneri using SDS-PAGE

SDS-PAGE was used to determine the protein profiles of *S. flexneri.* Proteins were separated on 10% polyacrylamide gel with a 4% stacking gel on top. Approximately 30 μg of OMPs from *S. flexneri* ATCC 12022 and clinical isolate SH057 expressed at 37, 38.5, and 40°C were loaded in each well and profiled using PROTEAN II xi (Bio-Rad). The OMPs obtained were resuspended with sample buffer containing 0.1% SDS and 2-β-mercaptoethanol (10%), heated at 100°C for 10 minutes, and electrophoresed at room temperature at constant current (30 mA) for 4 hours. The OMP profiles of *S. flexneri* were observed via SDS-PAGE gels stained with Coomassie brilliant blue (Bio-Rad). Molecular weight protein standards (Amersham, UK) were used for molecular weight determination. The molecular weights of OMPs were determined from the SDS-PAGE gels using an image analyser (SYNGENE Bio Imaging System, Japan).

### MALDI-ToF-ToF Mass Spectrometry

Three protein bands (18.4, 25.6, and 57.0 kDa) that showed a prominent increase in expression at 40°C were identified by performing MALDI-ToF-ToF. The OMPs of SH057 were resolved on 10% SDS-PAGE gel using the Mini PROTEAN 3 system (Bio-Rad). Approximately 100 μg of OMPs were loaded for the preparative comb gel (Bio-Rad). The gel was stained with Coomassie brilliant blue R220 to visualize the protein profile. The targeted protein bands were carefully excised from the preparative gel with a surgical blade as a narrow sharp band to avoid background contamination. The excised protein bands were kept in sterile 1.5 ml tubes and sent to Proteomics International Pty Ltd. (Nedlands, Western Australia) for further analysis. Briefly, the protein samples were first digested with mass spectrometry grade trypsin, and the peptides were extracted according to standard techniques. The protein bands were subjected to mass spectrometry analysis in which the peptides were analysed using a MALDI-ToF-ToF mass spectrometer (ABI 4800 instrument). The identified peptide fragments were searched against Mascot, Matrix Science (http://www.matrixscience.com). The search parameters were as follows: trypsin digestion with one missed cleavage; carbamidomethyl modification of cysteine as a fixed modification and oxidation of methionine as a variable modification; peptide tolerance, ± 0.6; MS/MS tolerance, ± 0.6; peptide charge, 1+; monoisotopic mass.

## Results

### The OMP profiles of S. flexneri ATCC 12022 and clinical isolate SH057 expressed at 37, 38.5, and 40°C

**Figure 1:**
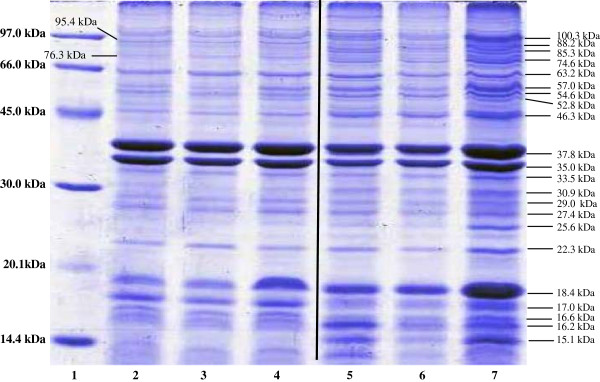
**SDS-PAGE gel showing the outer membrane protein (OMP) profiles of *S. flexneri *** **ATCC 12022 and the clinical isolate SH057 at 37°C, 38.5°C, and 40°C.**

Figure 1 shows the OMP profiles of ATCC 12022 and clinical isolate SH057 expressed at 37, 38.5, and 40°C. OMPs were detected in both strains at the tested temperatures, and a total of 24 protein bands were observed in the SDS-PAGE (Table 1). Sixteen protein bands were expressed by both strains at the three temperatures tested. Of the 24 bands, 75% were present in ATCC 12022 and 88% were observed in SH057 at 37 and 38.5°C. At 40°C, 79% and 92% of the proteins were detected in ATCC 12022 and the clinical isolate, respectively. A unique protein band (52.8 kDa) was expressed in both strains only at 40°C. Five protein bands (15.1, 25.6, 74.6, 85.3, and 88.2 kDa) were expressed by the clinical isolate at all three temperatures but were not expressed by ATCC 12022. Two protein bands (76.3 and 95.4 kDa) were only expressed by the ATCC strain at all three temperatures.

**Table 1:** **Overall expression of OMPs present in *S. flexneri *** **ATCC12022 and the clinical isolate SH057 at 37°C, 38.5°C, and 40°C**

**Band no.**	**Molecular weight (kDa)**	**ATCC 12022**			**SH057**		
		**(37°C)**	**(38.5°C)**	**(40°C)**	**(37°C)**	**(38.5°C)**	**(40°C)**
1	15.1	×	×	×	√	√	√
2	16.2	√	√	√	√	√	√
3	16.6	√	√	√	√	√	√
4	17.0	√	√	√	√	√	√
5	18.4	√	√	√	√	√	√
6	22.3	√	√	√	√	√	√
7	25.6	×	×	×	√	√	√
8	27.4	√	√	√	√	√	√
9	29.0	√	√	√	√	√	√
10	30.9	√	√	√	√	√	√
11	33.5	√	√	√	√	√	√
12	35.0	√	√	√	√	√	√
13	37.8	√	√	√	√	√	√
14	46.3	√	√	√	√	√	√
15	52.8	×	×	√	×	×	√
16	54.6	√	√	√	√	√	√
17	57.0	√	√	√	√	√	√
18	63.2	√	√	√	√	√	√
19	74.6	x	x	x	√	√	√
20	76.3	√	√	√	x	x	x
21	85.3	x	x	x	√	√	√
22	88.2	x	x	x	√	√	√
23	95.4	√	√	√	x	x	x
24	100.3	√	√	√	√	√	√

### Comparison of the expression levels of OMPs in S. flexneri ATCC 12022 and clinical isolate SH057

The degrees of OMP expression in both strains at 38.5 and 40°C were compared to those at 37°C and were found to differ (Table 2). For example, the level of the 30.9 kDa protein band was maintained in the ATCC strain at 40°C (same level of expression as seen in the 37°C), whereas it showed increased expression in the clinical isolate. The level of the 57.0 kDa protein band also was maintained in the ATCC strain at 38.5°C, but it was decreased in the clinical isolate. However, this protein’s expression was increased at 40°C in both strains. The expressions of the 18.4, 30.9, and 100.3 kDa proteins were maintained at 38.5°C but up-regulated at 40°C in both strains. There also was a prominent increase in the expression of the 18.4 kDa protein at 40°C in both strains. Overall, the majority of proteins in the clinical isolate were found to be up-regulated at 40°C, whereas six proteins (15.1, 16.2, 25.6, 57.0, 63.2, and 85.3 kDa) showed decreased expression at 38.5°C. Fourteen of the 18 proteins found in the ATCC strain were insensitive to the increased temperature.

**Table 2:** **Expression of OMPs in the ATCC and SH057 strains of *S. flexneri *** **at 38.5°C and 40°C in comparison to that at 37°C**

**Band no.**	**Molecular weight (kDa)**	**ATCC 12022(38.5°C)**	**ATCC 12022(40°C)**	**SH057(38.5°C)**	**SH057(40°C)**
1	**15.1**	**×**	**×**	↓	↑
2	**16.2**	→	→	↓	→
3	**16.6**	→	→	→	↑
4	**17.0**	→	→	→	↑
5	**18.4**	→	↓	→	↑
6	**22.3**	→	→	→	↑
7	**25.6**	**×**	**×**	↓	↑
8	**27.4**	→	→	→	→
9	**29.0**	→	→	→	→
10	**30.9**	→	→	→	↑
11	**33.5**	→	→	→	↑
12	**35.0**	→	→	→	↑
13	**37.8**	→	→	→	↑
14	**46.3**	→	↓	→	↑
15	**54.6**	→	→	→	↑
16	**57.0**	→	↓	↓	↑
17	**63.2**	→	→	↓	→
18	**74.6**	**×**	**×**	→	↑
19	**76.3**	→	→	**×**	**×**
20	**85.3**	**×**	**×**	↓	→
21	**88.2**	**×**	**×**	→	↑
22	**95.4**	→	→	**×**	**×**
23	**100.3**	→	↑	→	↑

↑ Increased expression of protein.

↓ Decreased expression of protein.

→ Same level expression of protein.

x: No protein band seen in one of the temperature.

The MALDI-ToF mass spectrometry analysis using Mascot (www. matrixscience.com) was performed to identify the proteins showing overexpression during heat stressed.

According to the Mascot search results obtained using the mass spectrometry database search engine, the 18.4 kDa protein was a match for Dps (a DNA starvation/stationary phase protection protein) with a score of 537 with 4 matched peptides. A score of > 36 indicates identity or extensive homology at a significant level (p < 0.05). The 25.6 kDa protein was a matched for PepA, and the 57.0 kDa protein was a match for WrbA with only one matched peptide identified.

## Discussion

Due to their location on the cell, OMPs have been shown to elicit host immune responses and hence are categorized as virulence factors [18]. During entry into a host, pathogenic bacteria may experience many types of stresses, such as those caused by changes in temperature, pH, osmotic strength, and iron availability, and these factors greatly influence the growth of the pathogenic organisms. Temperature variation is one of the most important stress factors that can be used to demonstrate the presence of heat shock OMPs in *S. flexneri*.

We conducted this study to determine how *S. flexneri* responds at the proteomic level to the stress of elevated temperature. When temperature was increased to 40°C, several OMPs responded by either increasing or decreasing their expression. To avoid technical errors, several measures were taken to ensure that the observed up-regulation or down-regulation of OMPs expressed at different temperatures was due only to temperature and not to different amounts of bacterial cells obtained due to different growth conditions. Before the extraction procedure, the concentration of bacterial cells at different growth conditions was normalized to OD_600_ 1.8, based on the growth curve analysis and total bacteria count performed. Furthermore, the same quantities of OMPs were used for analysis of the ATCC and SH057 strains expressed at 37, 38.5, and 40°C. The protein extraction at all three temperatures was repeated at least twice to ensure reproducibility and that consistent protein profiles were seen.

A prominent increase in expression of the 18.4 kDa protein was observed at 40°C in both strains, and overall, most of OMPs in clinical isolate the clinical OMPs shown increased expression at higher temperature at 40°C. This increase in protein expression at higher temperature seems to be an immediate response to stress; it might serve as a protective or survival mechanism by making as much of the essential proteins as possible under adverse conditions to ensure cell growth [19]. These proteins must be important for cell survival because they were expressed more during heat stress. A similar comparative study at different temperatures was conducted in China to investigate the protein expression profile of *S. flexneri* at 30 and 37°C. At 37°C the expressions of most of the virulence-related proteins were up-regulated, including IpaA, IpaB, IpaC, and IpaD [20]. In our study, temperatures higher than 37°C were chosen to investigate the proteome profile of the bacteria during enteric infection, as patients infected with *Shigella* spp. usually have a low to medium grade fever ranging from 38 to 40°C.

Protein expression at 40°C might be related to the virulence of the pathogen. Bacteria possess specific sensors that respond to stimuli from their new environment, which enable them to express the virulence factor only when required [21]. There are broad ranges of stimuli that bacteria sense and to which they respond. These stimuli include changes in pH, PO2, osmolarity, and temperature. For example, in *Vibrio cholerae,* activation of virulence factors by Tox R, a virulence regulon, is influenced by external stimuli such as temperature, osmolarity, and pH. Several previous studies described the expression of virulence genes in bacteria in response to temperature changes. These include expression of the regulatory gene virF in *Yersinia* spp. that controls the expression of the OMP Yop, which is related to virulence of the pathogen, and the regulatory gene prfA in *L. monocytogens* that regulates the expression of listeriolysin, which is a virulence factor that helps the survival of the bacterium [22,23].

In the ATCC strain, the expression profiles of most of the proteins did not change with increasing temperature. The maintenance of a constant expression level of some proteins at 38.5 and 40°C may indicate that either they lack a role in the survival and virulence of the organism or that the synthesis of these proteins is necessary to protect cells from the detrimental effects of stress stimuli (e.g., increased temperature, exposure to oxygen radicals, or nutritional deficiencies), [24]. Further studies are needed to elucidate the role of these proteins in the virulence and survival of the pathogen.

Dps have been identified in *Escherichia coli* as a low molecular weight protein that accumulates during the stationary phase and binds to DNA [25]. Moreover, Dps protein was found to be highly up-regulated in *E. coli* cells as temperature increased [26]. Luders *et al.*[26] reported that the increase in temperature induces the synthesis of more oxygen radicals, and the cells need to protect their cellular components from heat stress. Thus, overexpression of the dps gene is one of the protective mechanisms that is used during heat stress. dps also plays a major role in protecting genomic DNA against oxidative stress [27], nuclease cleavage, UV light, and thermal stress by its ability to bind with DNA to block the stress elements that attack DNA [28]. Halsey *et al.*[29] described the role of Dps in oxidative stress resistance and virulence in *Salmonella enterica* serovar Typhimurium. They demonstrated the ability of Dps to protect *Salmonella* from oxidative stress during infection, which enhanceed the virulence of the pathogen. In the current study, the increased expression of the 18.4 kDa protein (which was identified as Dps) at 40°C by *S. flexneri* might be an adaptive response of the bacterium to the heat stress environment.

WrbA, which was been studied most extensively in *E.coli*, is member of the highly conserved family of proteins involved in the cellular response to altered redox conditions and to different kinds of stress [30]. It has sequence similarity to proteins involved in quinonereductase activity [31,32]. WrbA protein is also known as TrpR binding protein, due to its ability to co-purify and co-immunoprecipitate with the tryptophan repressor protein TrpR [33]. Recently, Wang *et al.*[34] demonstrated that WrbA is one of the target proteins for the salicylidene acyl hydrazides, which are compound that can block the virulence of many Gram negative pathogens. Furthermore, Wang *et al.* reported that WrbA also contributed to the normal regulation and expression of virulence factors, especially the Type 3 secretion system and the bacterial flagella.

PepA is a member of the leucylaminopeptidase family of metallopeptidases [35]. In *E. coli*, PepA has been shown to be an active leucineaminopeptidase [36] that plays an important role in protein degradation and metabolism of biologically active peptides [37]. Luders *et al.* demonstrated that several enzymes involved in amino acid biosynthesis were up-regulated in *E. coli* cells as temperature increased, including PepA [26]. PepA was reported to be a strong thermostable protein that is able to degrade proteins [38]. In the current study, many proteins were synthesized and degraded by the bacterial cells when they were exposed to high temperatures to adapt the stress conditions. Thus, the up-regulation of PepA at 40°C seen in both strains likely allowed the bacterial cells to synthesize or degrade new proteins in order to adapt to the heat stress environment.

In this study, the proteome profiles of *S. flexneri* cells grown at 37, 38.5, and 40°C were analysed to determine the expression levels of OMPs. The overexpression of certain proteins (18.4, 25.6, and 57.0 kDa) when cells were exposed to higher temperatures likely was the pathogen’s strategy to regulate expression of virulence-related proteins. These proteins were identified by mass spectrometry as being HSPs (Dps and PepA) and a virulence-associated protein (WrbA). This is the first report describing the occurrence of these temperature-regulated proteins in *S. flexneri*. Further studies are needed to elucidate the role of these proteins in the survival and virulence of the organism at higher body temperatures during infection.

## Competing interests

The authors declare that they have no competing interests.

## Authors’ contributions

HH carried out the experiments and drafted the manuscript. AI participated in the acquisition of funding and in study design. KBS participated in the acquisition of funding and coordination and monitoring of research. Both AI and KBS also edited the manuscript. All authors have read and approved the final manuscript.
